# Immunohistochemical Evidence from APP-Transgenic Mice for Glutaminyl Cyclase as Drug Target to Diminish pE-Abeta Formation

**DOI:** 10.3390/molecules23040924

**Published:** 2018-04-17

**Authors:** Maike Hartlage-Rübsamen, Alexandra Bluhm, Anke Piechotta, Miriam Linnert, Jens-Ulrich Rahfeld, Hans-Ulrich Demuth, Inge Lues, Peer-Hendrik Kuhn, Stefan F. Lichtenthaler, Steffen Roßner, Corinna Höfling

**Affiliations:** 1 Paul Flechsig Institute for Brain Research, University of Leipzig, 04103 Leipzig, Germany; maikerbs@rz.uni-leipzig.de (M.H.-R.); alexandra.bluhm@medizin.uni-leipzig.de (A.B.); 2 Department of Molecular Drug Design and Target Validation, Fraunhofer Institute for Cell Therapy and Immunology, 06120 Halle (Saale), Germany; anke.piechotta@izi.fraunhofer.de (A.P.); miriam.linnert@izi.fraunhofer.de (M.L.); jens-ulrich.rahfeld@izi.fraunhofer.de (J.-U.R.); Hans-Ulrich.Demuth@izi.fraunhofer.de (H.-U.D.); 3 Probiodrug AG, 06120 Halle (Saale), Germany; Inge.lues@probiodrug.de; 4 Institute of Pathology, Technical University of Munich, 81675 Munich, Germany; Peer.Kuhn@tum.de; 5 Deutsches Zentrum für Neurodegenerative Erkrankungen (DZNE), 81377 Munich, Germany; stefan.lichtenthaler@dzne.de; 6 Munich Cluster of Systems Neurology (SyNergy), 81377 Munich, Germany; 7 Neuroproteomics, School of Medicine, Klinikum rechts der Isar, Technical University of Munich, 81675 Munich, Germany; 8 Institute for Advanced Study, Technical University of Munich, 85748 Garching, Germany

**Keywords:** Alzheimer’s disease, animal model, human APP expression, glutaminyl cyclase, pyroglutamate-Abeta

## Abstract

Oligomeric assemblies of neurotoxic amyloid beta (Abeta) peptides generated by proteolytical processing of the amyloid precursor protein (APP) play a key role in the pathogenesis of Alzheimer’s disease (AD). In recent years, a substantial heterogeneity of Abeta peptides with distinct biophysical and cell biological properties has been demonstrated. Among these, a particularly neurotoxic and disease-specific Abeta variant is N-terminally truncated and modified to pyroglutamate (pE-Abeta). Cell biological and animal experimental studies imply the catalysis of this modification by the enzyme glutaminyl cyclase (QC). However, direct histopathological evidence in transgenic animals from comparative brain region and cell type-specific expression of transgenic hAPP and QC, on the one hand, and on the formation of pE-Abeta aggregates, on the other, is lacking. Here, using single light microscopic, as well as triple immunofluorescent, labeling, we report the deposition of pE-Abeta only in the brain regions of APP-transgenic Tg2576 mice with detectable human APP and endogenous QC expression, such as the hippocampus, piriform cortex, and amygdala. Brain regions showing human APP expression without the concomitant presence of QC (the anterodorsal thalamic nucleus and perifornical nucleus) do not display pE-Abeta plaque formation. However, we also identified brain regions with substantial expression of human APP and QC in the absence of pE-Abeta deposition (the Edinger-Westphal nucleus and locus coeruleus). In these brain regions, the enzymes required to generate N-truncated Abeta peptides as substrates for QC might be lacking. Our observations provide additional evidence for an involvement of QC in AD pathogenesis via QC-catalyzed pE-Abeta formation.

## 1. Introduction

It is generally accepted that increased amyloidogenic processing of the amyloid precursor protein (APP), compromised amyloid beta (Abeta) degradation, and post-translational modifications of Abeta peptides contribute to the pathogenesis of Alzheimer’s disease (AD) [1,2]. In particular, specific post-translational Abeta modifications were shown to increase its hydrophobicity, aggregation propensity, and neurotoxicity and to compromise its proteolytical degradation, which collectively promote the formation of high molecular weight Abeta aggregates and AD pathogenesis. Such pathogenic post-translational Abeta modifications include tyrosine10-Abeta nitration, which is catalyzed by nitric oxide synthase 2 (NOS2) and thus can be prevented by the NOS2 inhibitor L-NIL and by genetic NOS2 ablation [3]. Additionally, the phosphorylation of serine8- and serine26-Abeta was shown to occur via different pathways and to have diverse effects on the formation of Abeta aggregates [4,5,6,7]. Furthermore, a number of N-terminal Abeta truncations have been reported [8,9,10,11,12,13]. These N-terminally truncated Abeta peptides account for the majority of Abeta peptides in the AD brain but not in the brains of APP-transgenic mice generated to mimic aspects of AD pathology [14,15].

**Figure 1:**
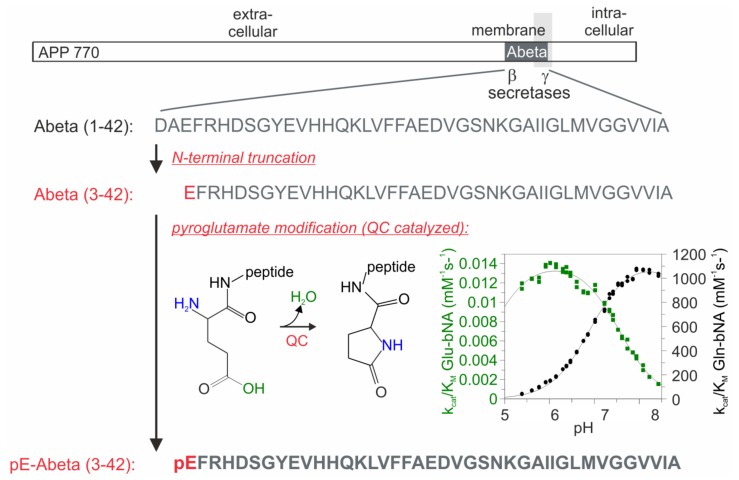
Sequential generation of pE-Abeta peptides. Abeta(1–42) peptides are generated by β-secretase and γ-secretase cleavage of amyloid precursor protein (APP). After N-terminal removal of two amino acids, a glutamate residue (E, red) is exposed at position 3 of Abeta(3–42) and can be converted by the enzymatic activity of glutaminyl cyclase (QC) to pE resulting in the peptide pE-Abeta(3–42). The enzymatic reaction of pE formation and kinetic characteristics are shown in the bottom figure. Note the 5-oxoproline ring formation under liberation of water (left) and the slow conversion of N-terminal glutamate under slightly acidic pH conditions (green curve), as compared with the much faster pE formation from N-terminal glutamine (black curve; right). Enzymatic presentations are adapted from Schilling et al. [18].

N-terminally truncated Abeta peptides at positions 3 and 11 may serve as substrates for subsequent pyroglutamate (pE)3- and pE11-Abeta modifications [16,17]. However, for about a decade, the mechanism of pE-Abeta generation remained elusive. Schilling et al. [18] provided the first evidence from substrate conversion in vitro that the enzyme glutaminyl cyclase (QC) acts as glutamyl cyclase to catalyze pE-Abeta formation from N-terminal glutamate (Figure 1). Subsequently, QC was detected in human brain regions displaying pE-Abeta immunoreactivity, such as the Edinger-Westphal nucleus and locus coeruleus [19], hippocampus [20], and neocortex [21]. Additionally, chronic pharmacological inhibition [22] or genetic ablation [23,24] of QC in transgenic mouse and drosophila models of AD resulted in reduced pE-Abeta peptide generation and in ameliorated behavioral deficits, while QC overexpression aggravated neuropathology and cognitive dysfunction in transgenic mice [24]. pE-Abeta peptides act as seeds for the co-aggregation of unmodified Abeta peptides [25] and transmit their structural and neurotoxic properties in a prion-like fashion [26].

In APP-transgenic experimental AD animal models, however, the proportion of pE-Abeta on total Abeta was reported to be much lower than in the human brain [27]. Additionally, the lack of specific antibodies to detect transgenic hAPP expression and the questionable performance of commercial QC antibodies so far did not allow us to directly relate the expression of both proteins to pE-Abeta formation in transgenic mice.

Using the rabbit anti-QC antiserum 1301, tested to be specific in QC knock-out mouse brain [28,29], we observed robust QC expression in the hypothalamic nuclei, where physiological QC substrates reside. Additionally, a subpopulation of neocortical and of GABAergic interneurons in the mouse hippocampus displays QC immunoreactivity [28]. Moreover, we demonstrated pronounced QC immunoreactivity in subcortical mouse brain regions that are vulnerable to AD, such as nucleus basalis, locus coeruleus, and Edinger-Westphal nucleus [19]. Here, we introduce a novel goat anti-QC antiserum with similar specificity characteristics and staining patterns in mouse brain to the rabbit antiserum 1301, which provides sustainable resources for future studies.

Additionally, the recently established rat monoclonal antibody 1D1, differentiating between human and mouse APP, allows for specific mapping of hAPP transgene expression patterns in the brains of APP-transgenic mice [30]. We also introduce a novel pE-Abeta-specific mouse monoclonal antibody, which does not cross-react either with the intact N-terminus of Abeta or with isoAsp7-modified Abeta peptides and binds to pE-Abeta with high affinity (Kd = 1.6 nM). In the present study, we combined those tools for a comparative analysis of spatial hAPP and QC expression with pE-Abeta formation in the brains of APP-transgenic Tg2576 mice. Our data indicate that pE-Abeta is only generated at sites of complementary hAPP and endogenous QC expression and that QC is a suitable drug target for AD treatment.

## 2. Results

### 2.1. Specificity of the Rat Anti-hAPP, Goat Anti-Mouse QC and Mouse Anti-pE-Abeta Antibodies

The specificity of the antibodies used in this study is critical for the interpretation of the results. Therefore, antibodies raised against hAPP (from rat, clone 1D1) and against mouse QC (from goat) were tested in mouse brain tissue lacking the respective antigens as negative controls. The specificity of the monoclonal pE-Abeta antibody (from mouse, clone J8) was tested on dot blots.

**Figure 2:**
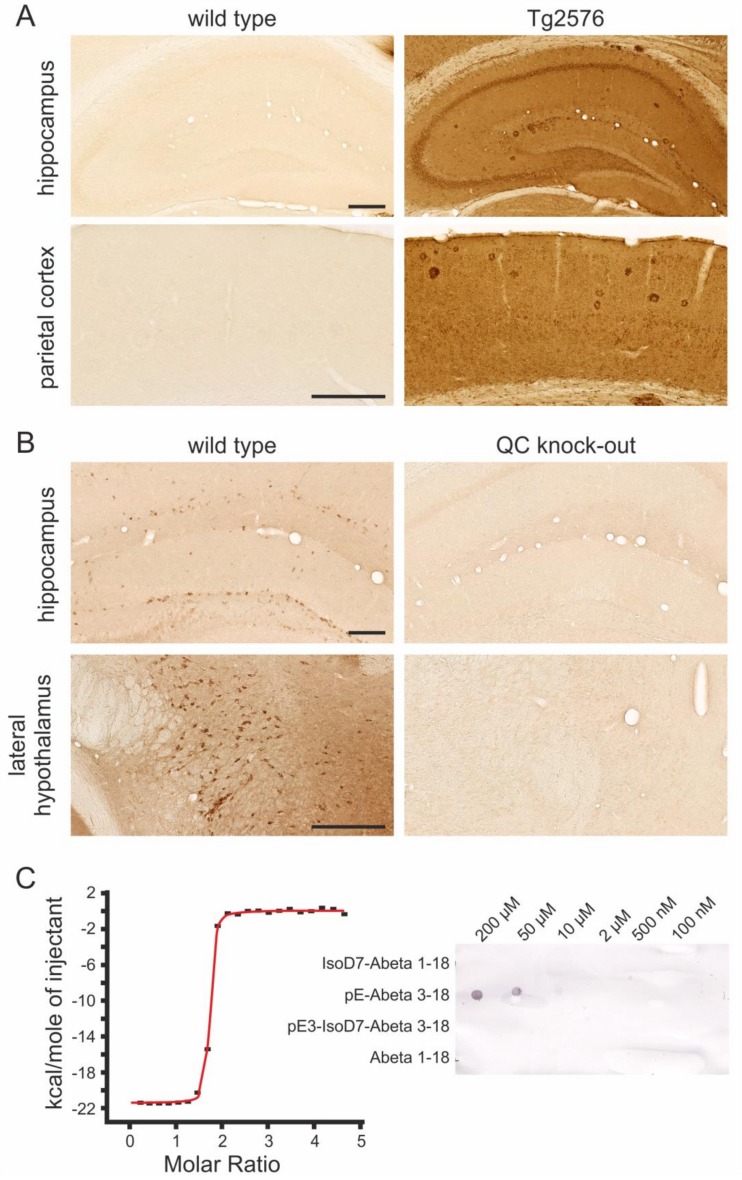
Demonstration of the specificity of antibodies used in this study. (**A**) The monoclonal rat anti-hAPP antibody 1D1 labeled neurons and amyloid plaques in hAPP-transgenic Tg2576 mouse brain as shown for the hippocampus and parietal cortex (**right**), whereas this labeling was lacking in corresponding wild type mouse brain sections (**left**). (**B**) The specificity of the goat anti-QC antiserum is demonstrated by marked labeling of hippocampal interneurons and of neurons in the lateral hypothalamus of wild type mice (**left**), which is absent in QC knock-out mouse brain sections (**right**). Scale bars in (**A**,**B**) represent 200 μm. (**C**) The mouse monoclonal anti-pE-Abeta antibody J8 detects pE-Abeta3-18, but not Abeta1-18 or isoAsp7-modified Abeta peptides spotted onto nitrocellulose membranes by dot blot analysis (**right**). Isothermal titration calorimetry revealed a K_d_ value of 1.6 nM for J8 towards pE-Abeta (**left**).

In agreement with our recent study [30], the rat anti-hAPP antibody 1D1 only labeled neurons in hAPP-transgenic Tg2576 mice, but not in wild type littermates (Figure 2A). Similarly, in QC knock-out mice, no neuronal labeling was generated by the goat anti-mouse QC antiserum (Figure 2B), and the staining pattern in wild type mice corresponded to the one obtained by the rabbit anti-QC antiserum 1301, which has been used in several preceding studies [19,20,28,29,31]. Furthermore, dot blot analysis of unmodified Abeta and pE-Abeta peptides spotted onto nitrocellulose membranes revealed the detection of pE-Abeta, but not of full length Abeta, by the J8 monoclonal antibody (Figure 2C).

### 2.2. Spatial Relation of hAPP and QC Expression with pE-Abeta Deposition in Tg2576 Mouse Brain 

In order to obtain insights into the interrelation of the spatial appearance of hAPP, QC, and pE-Abeta, single immunohistochemical screenings for the three antigens were performed in Tg2576 mouse coronal brain sections. Since the pE-Abeta pathology is only present at an advanced age, 18-month-old mice were used for the analyses of transgenic hAPP expression and pE-Abeta pathology. On the other hand, the expression of endogenous QC declines during aging [28] and was, therefore, studied at the postnatal age of 3 months.

**Figure 3:**
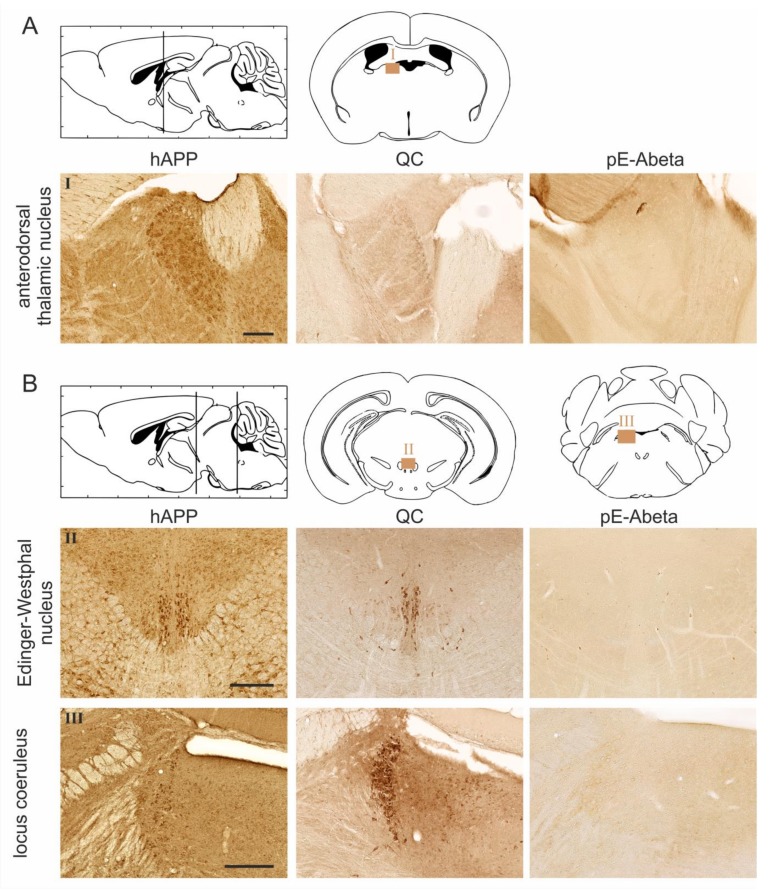

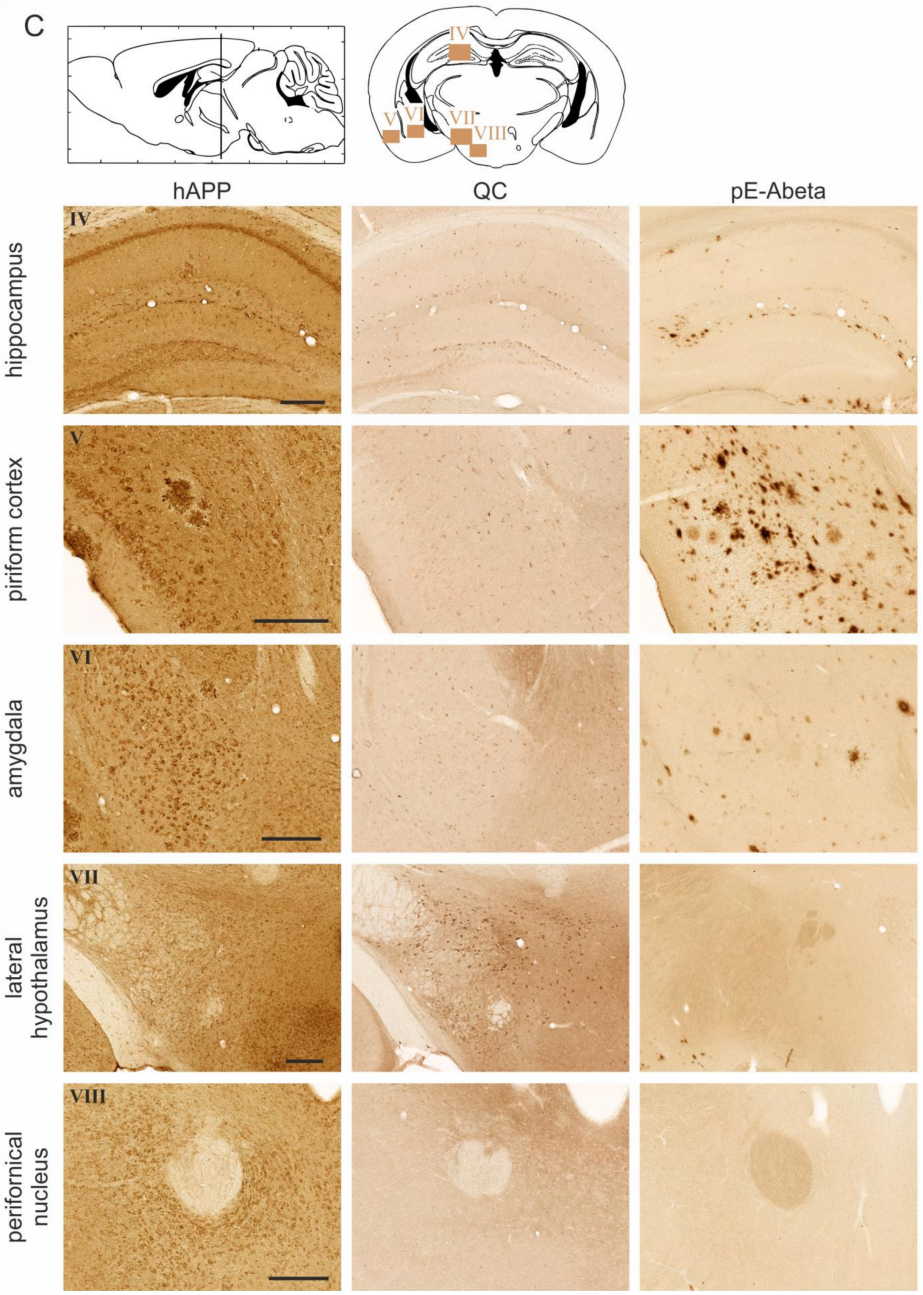
Immunohistochemical labelings for hAPP, QC, and pE-Abeta in Tg2576 mouse brain. The presence of hAPP, endogenous QC, and pE-Abeta was analyzed at four coronal brain cutting levels as indicated in the schematic sagittal brain sections. In the schematic coronal brain sections, the areas of interest are marked with light brown squares and Roman numerals (I to VIII) to allow identification of the immunohistochemical images. Mice at the age of 18 months were used to monitor hAPP transgene expression and pE-Abeta pathology. The expression of endogenous QC was analyzed in three-month-old mice. In the anterodorsal thalamic nucleus, (**A**) hAPP is expressed, while QC and pE-Abeta were not detected, consistent with QC being a prerequisite for pE-Abeta formation. In the Edinger-Westphal nucleus and locus coeruleus (**B**), both hAPP and QC are highly abundant; however, pE-Abeta pathology is absent. In other brain regions with substantial hAPP and QC expression (hippocampus, piriform cortex, and amygdala), pE-Abeta deposits were present. In contrast, in brain regions with high hAPP and low QC expression (perifornical nucleus) or with low hAPP and high QC expression (lateral hypothalamus), no pE-Abeta pathology was detected (**C**). Scale bars in (**A**–**C**) represent 200 μm.

In Tg2576 mice, the transgenic hAPP is predominantly expressed by neurons in the neocortex, amygdala, Edinger-Westphal nucleus, locus coeruleus, anterodorsal thalamic nucleus, and perifornical nucleus (Figure 3A–C). In the lateral hypothalamus, hAPP expression was much weaker, and in the hippocampus, layer-specific transgene expression patterns were detected. In particular, hAPP was highly abundant in CA1 pyramidal neurons and in subpopulations of interneurons but absent from dentate gyrus granule cells (Figure 3C).

The Abeta-modifying enzyme QC is endogenously expressed by subsets of interneurons but not by pyramidal or granule cells of the hippocampus (Figure 3C). QC was also found to be abundantly expressed by neurons of the Edinger-Westphal nucleus and locus coeruleus (Figure 3B), where pathogenic functions of QC were reported, and by magnocellular neurons of the lateral hypothalamus, where physiological QC substrates are present (Figure 3C). In the piriform cortex and amygdala, QC was found to be expressed at moderate levels (Figure 3C).

Amyloid plaques containing pE-Abeta peptides generated by the enzymatic activity of QC on N-terminally truncated Abeta peptides derived from hAPP were specifically detected in the neocortex, hippocampus, and amygdala (Figure 3C) but not in subcortical structures, such as the anterodorsal thalamic nucleus (Figure 3A), Edinger-Westphal nucleus, locus coeruleus (Figure 3B), lateral hypothalamus, and perifornical nucleus (Figure 3C). 

The relative abundance of hAPP, endogenous QC, and pE-Abeta is summarized in Table 1. Here, it becomes obvious that pE-Abeta in the brains of Tg2576 mice only arises in brain structures with hAPP and endogenous QC co-expression and not in brain regions where one of these proteins is lacking. On the other hand, the presence of both proteins—although required—is not sufficient for pE-Abeta formation.

**Figure 4:**
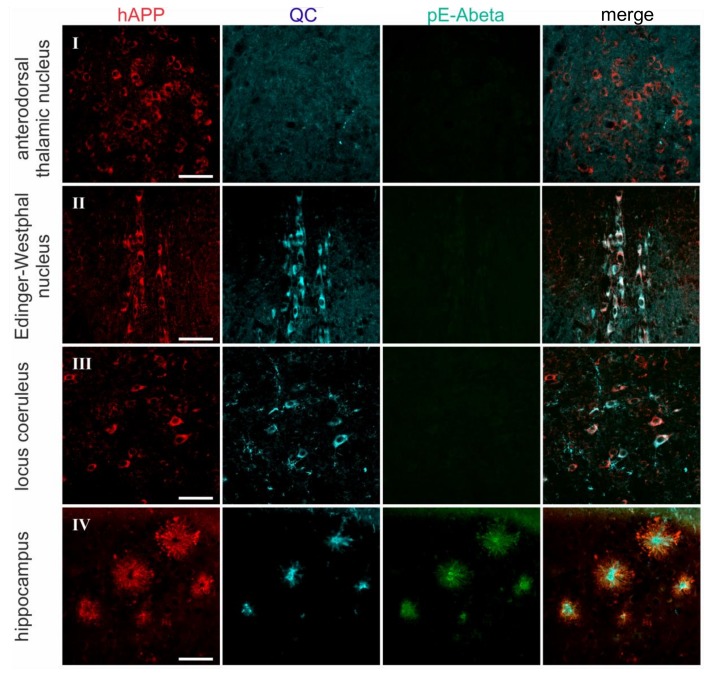

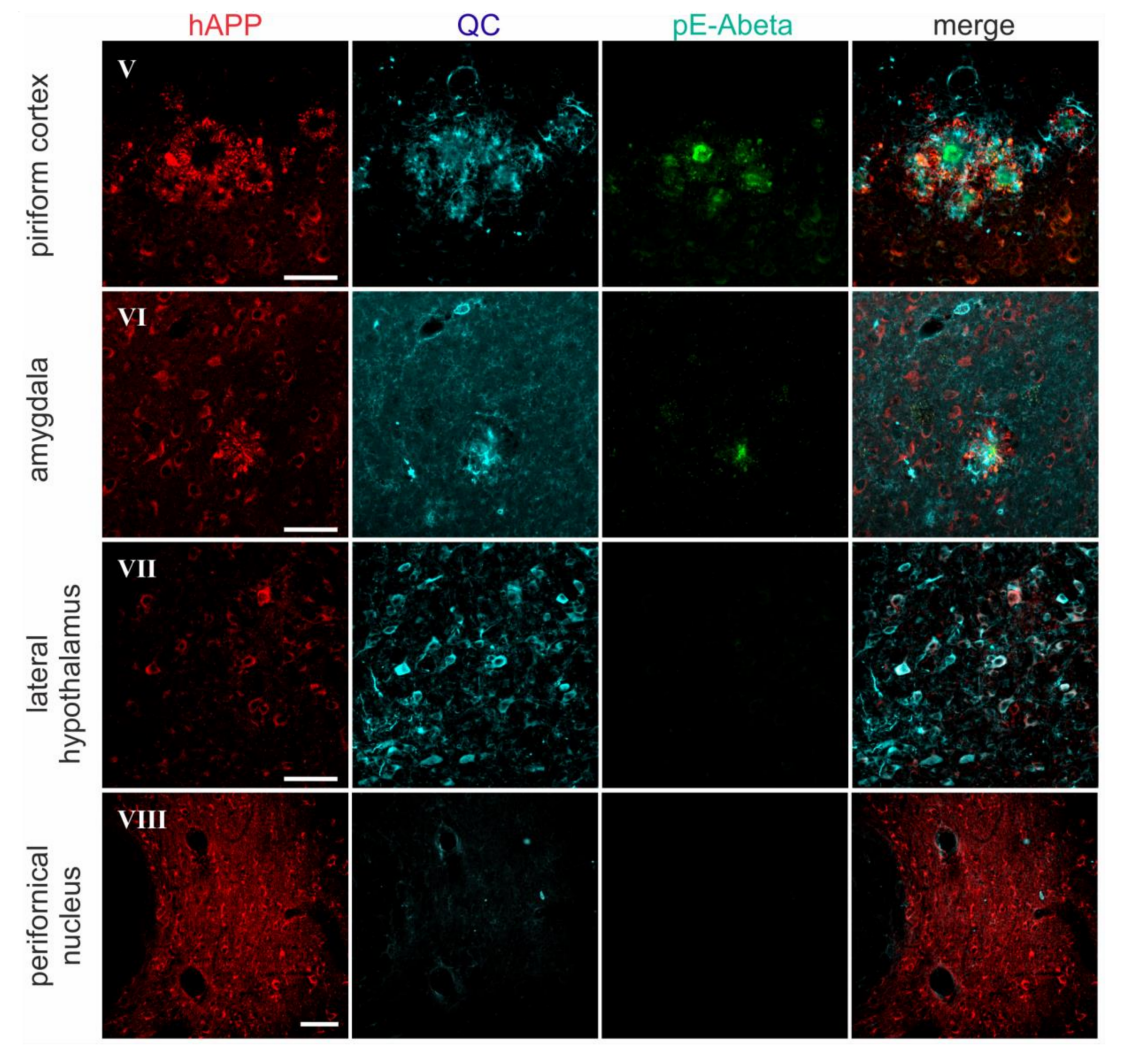
Triple immunofluorescent labeling of hAPP, QC, and pE-Abeta. hAPP, QC, and pE-Abeta were visualized by triple immunofluorescent labeling with secondary antibodies from donkey resulting in red (hAPP), blue (QC), and green (pE-Abeta) labeling. Roman numerals I-VIII correspond with the numbers in Figure 3 and indicate the areas of interest. Scale bars represent 50 μm.

The rat, goat, and mouse origin of the hAPP, QC, and pE-Abeta antibodies used in this study allowed their combination in triple immunofluorescent labeling to directly relate the spatial appearance of all three proteins. A co-localization of the three target proteins was only detected in the hippocampus, piriform cortex, and amygdala (Figure 4). This is consistent with single labeling data and supportive of QC being a prerequisite for pE-Abeta generation. Brain regions lacking QC expression (anterodorsal thalamic nucleus and perifornical nucleus) did not display pE-Abeta aggregates in immunofluorescent labeling (Figure 4). However, there are brain regions devoid of pE-Abeta pathology in spite of substantial hAPP and QC expression (Edinger-Westphal nucleus, locus coeruleus, and lateral hypothalamus; Figure 4).

## 3. Discussion

Here, we demonstrate a close histopathological association between the spatial co-expression of hAPP and the enzyme QC, on the one side, and the deposition of pE-Abeta peptides in the brains of APP-transgenic Tg2576 mice, on the other. A role for QC in the pE modification of N-terminally truncated Abeta peptides was first suggested in cell-free assays using purified QC and peptide substrates by Schilling et al. [18]. In contrast to N-terminal glutamine residues, which spontaneously convert to pE, N-terminal glutamate residues require enzymatic QC activity for pE conversion [32]. Substantial evidence to support this notion was built up in cell biological and animal experimental studies. In particular, QC was shown to catalyze pE-formation from N-terminal glutamate and glutamine precursors in cultured cells, and QC inhibition suppressed pE-Abeta formation [33,34]. In transgenic AD models, both pharmacological inhibition or genetic ablation of QC resulted in reduced pE-Abeta peptide generation [22,23,24], whereas QC overexpression aggravated neuropathology and cognitive dysfunction [24].

In human brain tissue from AD subjects, we demonstrated a tight spatial co-occurrence between QC and the intracellular and extracellular formation of pE-Abeta aggregates in the Edinger-Westphal nucleus, locus coeruleus, nucleus basalis Meynert [19], and neocortex [21]. In the hippocampus of AD subjects, the formation of focal pE-Abeta deposits was found to co-localize with QC expressing neurons, whereas extracellular diffuse pE-Abeta aggregates originated in terminal fields of projections arising from entorhinal cortex layer II neurons [20].

In APP-transgenic Tg2576 mice, however, pE-Abeta deposits concentrate in the neocortex and hippocampus and were not detected in the respective subcortical structures affected by this pathology in AD brains. These divergent findings could question the role of QC in pE-Abeta generation and/or point towards limitations of transgenic mouse models to mimic the complete spectrum of Abeta pathology in AD. An obvious explanation, however, is the lack of transgenic hAPP and/or endogenous QC expression in these subcortical brain structures. To address this question, specific experimental tools (i.e., antibodies discriminating transgenic hAPP expression from that of endogenous mouse APP and for the specific detection of QC and pE-Abeta) are required. Only recently, a hAPP-specific antibody meeting these criteria was established [30]. In addition, we here introduce a QC-specific antibody raised in goat and a novel pE-Abeta-specific antibody, which can be applied in immunohistochemical studies. Employing these novel antibodies, we demonstrate the generation of pE-Abeta deposits in brain regions, such as the piriform cortex, hippocampus, and amygdala, which express both hAPP and endogenous QC. In no case were pE-Abeta deposits detected in brain regions which lacked substantial QC expression. Thus, QC indeed appears to be a prerequisite for pE-Abeta formation. Interestingly, pE-Abeta plaque load is highest in the piriform cortex, although hAPP and QC expression there were rated comparable to that of the amygdala and hippocampus where plaque load is significantly lower (see Table 1). However, endogenous QC in the piriform cortex is mostly present in small interneurons that are not as noticeable as larger QC-positive neurons in other brain areas, and projections from QC-rich regions to the piriform cortex (e.g., neocortical areas and the entorhinal cortex, respectively) might add to local QC generation but was not quantifiable by the immunocytochemistry performed here. Moreover, there might be additional factors contributing to the accumulation of pE-Abeta plaques. For example, enzymes generating N-truncated Abeta peptides as QC substrates and Abeta or pE-Abeta-degrading enzymes might be present at different concentrations in defined neuronal populations. Moreover, the capacity of local microglial cells to clear pE-Abeta might be brain region-specific.

However, we also identified the Edinger-Westphal nucleus and locus coeruleus as brain regions where both hAPP and endogenous QC are highly abundant, but pE-Abeta deposits are lacking. This observation might be explained by different scenarios: (i) The enzymes generating the N-truncated Abeta precursor from hAPP or from Abeta(1–x), which serves as QC substrate, are not expressed by these neurons. There are significant ongoing efforts from different groups trying to identify an alternative beta-secretase-cleaving hAPP after position 2 of the Abeta sequence or aminopeptidases acting at Abeta(1–x) peptides to remove two N-terminal amino acids. The analyses of these brain structures in comparison to the piriform cortex and hippocampus in differential proteomic or molecular biological approaches might, therefore, be instrumental to identify and/or validate such enzymatic activities. (ii) In addition, the low abundance of the beta-secretase BACE1 in the Edinger-Westphal nucleus and locus coeruleus might be responsible for an overall lack of Abeta production. (iii) Alternatively, the instant proteolytical degradation of Abeta(1–x) peptides by neprilysin or insulin-degrading enzyme could prevent the formation of N-truncated Abeta peptides as QC substrates, even in the presence of BACE1.

We also propose to use primary neuronal cell cultures derived from Tg2576 fetuses. These neuronal cultures would probably display robust expression of secreted APP and readily detectable levels of Abeta. This system from primary transgenic APP neurons might allow to investigate ex vivo changes in APP processing and Abeta metabolism specific to neuronal functions after experimental manipulation of QC activity.

Because of their seeding capacity to induce deposition of unmodified Abeta peptides and their neurotoxic properties, pE-Abeta peptides appear as rational targets for AD therapy. Both prevention of pE-Abeta formation by inhibition of QC [22] and removal of pre-existing pE-Abeta aggregates by immunization [35,36,37,38] have been demonstrated to be effective therapeutic strategies in transgenic mouse models. In addition to pathogenic N-truncated Abeta peptides, there are also physiological QC substrates, such as neuropeptides and peptide hormones [39,40], that could be potentially affected by QC inhibition. However, in experimental animals with genetic QC ablation or pharmacological QC inhibition [41,42,43], it was found that the hypothalamic-gonadotropic and hypothalamic-thyroid axes, which are regulated by the QC substrates gonadoliberin (GnRH) and thyroliberin (TRH), are only marginally affected in presence of strong QC inhibition. Furthermore, in two clinical trials (see below), no effects on these hormone axes or other side effects with relation to QC substrates were observed, although a QC target occupancy in the brain of about 90% was reached under treatment. In general, virtually all physiological QC substrates carry N-terminal glutamine (Gln) residues, which can cyclize spontaneously—without QC activity—into pE, providing basal levels of pE-modified peptides. In contrast, the N-terminal glutamate (Glu) present at truncated Abeta peptides requires QC activity for conversion to pE-Abeta [32]. Moreover, the poor QC substrate Glu3-Abeta will be displaced from the enzyme more easily than high-affinity endogenous Gln substrates [43], resulting in specific inhibition of the pathogenic Glu3-Abeta substrate at inhibitor concentrations at which the physiological substrates are widely unaffected. Thus, blocking of pE-Abeta formation by QC inhibition appears to be a specific and safe therapeutic approach.

Indeed, a substantial number of QC inhibitors from different chemical classes have been identified and were further characterized [43,44,45]. One of these QC inhibitors, PQ912, was developed for application in human subjects in clinical trials. In a phase I clinical trial, the compound was well-tolerated in nonelderly and elderly human subjects, and a dose-dependent inhibition of QC activity in the spinal fluid was determined [46]. In a phase IIa clinical study, several encouraging results were obtained for various types of readouts: It was found that the AD-related alteration in the EEG power spectrum was changed towards normal, a clear indication of the reduction of the synaptic marker neurogranin and the inflammatory marker YKL-40—all of which are in support of protecting impaired synapses [47].

Taken together, our observations provide further evidence for the involvement of QC in AD pathogenesis by QC-catalyzed pE-Abeta formation. To consider the complex spectrum of enzymes involved in pE-Abeta generation, we suggest a brain region-specific differential expression analysis of alternative β-secretases and/or Abeta-truncating enzymes that generate N-truncated Abeta precursors for pE-Abeta formation by proteomic and molecular biological approaches.

## 4. Materials and Methods

### 4.1. APP-Transgenic Tg2576 Mice

In this study, the APP-transgenic Tg2576 mice developed and described earlier, were used as a model for amyloid pathology [48]. The mice contain hAPP695 with the Swedish double mutation (K670N, M671L) as transgene under control of a hamster prion protein promoter. Mice heterozygous for the transgene and wild type littermates were on a mixed C57BL/6 × SJL background. Mice were housed in groups of 3–5 animals per cage and separated by sex, with ad libitum access to water and food with 12 h day/12 h night cycles at 23 °C. The cages contained red plastic houses (Techniplast, Quakertown, PA, USA) and shredded paper flakes to allow nest building. At the age of six weeks, the transgenicity of the animals was tested by polymerase chain reaction of tail DNA, as described elsewhere [48]. Mice were studied at the age of 3 months for endogenous QC expression and at the age of 18 months for pE-Abeta pathology and transgenic hAPP expression. The rationale to use young mice for the analysis of QC distribution is the decline of QC expression in mouse brain during aging [28]. On the other hand, the late appearance of pE-Abeta pathology in Tg2576 mice only allows the monitoring of amyloid pathology at an advanced age [20]. Thus, in order to demonstrate the expression of all antigens at high levels, we decided to analyze two different postnatal ages, 3 and 18 months. We believe that this is justifiable because the occurrence of plaques in the brains of old Tg2576 mice is only the end of a progressive pathological process in which QC—according to this concept—plays a role from the very beginning. In all cases, age-matched, non-transgenic littermates served as controls. All experimental protocols were approved by the Landesdirektion Sachsen, license T28/16, and all methods were carried out in accordance with the relevant regulations and guidelines of the Federation of European Laboratory Animal Science Associations (FELASA).

### 4.2. Antibodies against hAPP, QC, and pE-Abeta 

To specifically detect transgenic hAPP, but not endogenous mouse APP, in Tg2576 mouse brain sections, we used the rat monoclonal antibody 1D1 [30] (see Figure 2A). The presence of mouse QC was detected using a novel goat anti-mouse QC antiserum. This antiserum generated the same staining pattern as that described for a rabbit QC antiserum [19,20,28,29,31] and did not yield labeling in brain sections from QC knock-out mice (Figure 2B). The localization of pE-Abeta immunoreactive plaques was revealed using the mouse monoclonal antibody J8. This antibody specifically detects the pE-Abeta neo-epitope generated by QC activity with high affinity (Kd = 1.6 nM) and does not cross-react with untruncated or isoAsp7-modified Abeta peptides as shown by isothermal titration calorimetry (ITC) and by dot blot analysis (Figure 2C). 

### 4.3. Isothermal Titration Calorimetry

The J8 antibody was dialyzed against an ITC buffer (150 mM NaCl, 25 mM Na_2_HPO_4_, 25 mM KH_2_PO_4_, 1 mM EDTA, pH 7.4) at 4 °C overnight. Measurements were performed at 25 °C using a VP-ITC MicroCalorimeter (MicroCal, Northampton, MA, USA). A 33.3 μM solution of the lyophilized pE3-18-Abeta peptide dissolved in an ITC buffer was injected in 21 cycles into the J8 antibody solution (1.67 μM) with a 5-min interval between injections. Binding enthalpies were corrected for dilution heat after titrating the peptide into the ITC buffer.

### 4.4. Dot Blot Analysis

Two μL of Abeta peptides were spotted in descending concentrations on a nitrocellulose membrane and blocked for 1 h in a blocking solution (5% (*w*/*v*) milk powder in TBS–T (TBS + 0.05% Tween 20 (*v*/*v*))). Antibody J8 was diluted to 1 μg/mL in a blocking solution and incubated with the membrane for 1 h, followed by 3 × 5 min washing steps with TBS–T. An anti-mouse antibody conjugated to alkaline phosphatase (AP) was added and incubated for 1 h, followed by 3 × 5 min washing steps and subsequent colorimetric detection of AP activity by the addition of substrates 5-bromo-4-chloro-3-indolyl-phosphate and nitro blue tetrazolium.

### 4.5. Immunohistochemistry

#### 4.5.1. Tissue Preparation 

Mice were sacrificed by CO_2_ inhalation and transcardially perfused with phosphate-buffered saline (pH 7.4) followed by 4% buffered paraformaldehyde through the left cardiac ventricle. After perfusion fixation, the brain was removed from the skull and placed in the same fixative overnight at 4 °C. After cryoprotection in 30% sucrose in 0.1 M phosphate buffer for 3 days, coronal sections (30 μm) were cut on a sliding microtome and collected in 0.1 M phosphate buffer containing 0.025% sodium azide.

#### 4.5.2. Single Labeling hAPP, QC, and pE-Abeta Immunohistochemistry

All immunohistochemical procedures were performed on free-floating brain sections. Brain sections were pre-treated with 1% H_2_O_2_ in 60% methanol for 1 h to abolish endogenous peroxidase activity. Unspecific staining was blocked in TBS containing 5% normal donkey serum and 0.3% Triton-X100 before incubating the brain sections with the primary antibodies against hAPP (rat anti-hAPP, clone 1D1, 1:4), against mouse QC (goat anti-mouse QC, 1:500), and against pE-Abeta (mouse anti-pE-Abeta, clone J8, 1:200), respectively, at 4 °C overnight. The following day, the sections were subsequently incubated with secondary, biotinylated donkey antibodies against rat, goat, and mouse IgG (Dianova; 1:1000) for 60 min at room temperature, followed by the ABC method, which was comprised of incubation with complexed streptavidin—biotinylated horseradish peroxidase. Incubations were separated by washing steps (3 × 5 min in TBS). Binding of peroxidase was visualized by incubation with 4 mg 3,3′-diaminobenzidine (DAB) and 2.5 μL H_2_O_2_ per 5 mL tris buffer (0.05 M; pH 7.6) for 3–5 min, resulting in brown labeling.

#### 4.5.3. Staging of Immunohistochemical Labeling

The immunohistochemical labeling with each antibody was performed simultaneously for all brain sections, which allowed comparison of the staining intensity across coronal levels and brain regions. The appearance of the immunohistochemical reaction was rated by three independent researchers blinded to the labeling for the number of neurons, staining intensity, and pE-Abeta plaque load in the given brain regions. This resulted in a six stage rating from “0” (absent) to “5” (highly abundant) for the three antigens analyzed.

#### 4.5.4. Triple Immunofluorescent Labeling

Simultaneous immunohistochemical labeling of hAPP, QC, and pE-Abeta in Tg2576 mouse brain sections was performed using a cocktail of rat monoclonal anti-hAPP (1D1, 1:2), goat anti-QC (1:250), and mouse monoclonal anti-pE-Abeta (J8, 1:100) antibodies. All sections were pre-treated with 60% methanol for 60 min, and unspecific staining was blocked by treatment with TBS containing 5% normal donkey serum and 0.3% Triton-X100 before incubating the brain sections with the primary antibody-mix in a blocking solution for 24 h at 4 °C. Thereafter, the brain sections were washed and transferred to a cocktail of secondary antibodies (i.e., biotin-conjugated donkey anti-rat (Dianova; 1:400), Cy2-conjugated donkey anti-mouse (Dianova; 1:200), and Cy5-conjugated donkey anti-goat (Dianova; 1:200)) IgGs in TBS containing 2% BSA for 60 min at room temperature. After washing, secondary biotinylated donkey anti-rat antibodies were marked by incubation with streptavidin–Cy3. The brain sections were then washed, mounted onto glass slides, and coverslipped.

#### 4.5.5. Light Microscopy

Tissue sections were examined with an Axio-Scan.Z1 microscope connected with a Colibri.7 light source and an Axiocam 506 (Carl Zeiss, Göttingen, Germany). Images were digitalized by means of ZEN 2.3 software and exported with the NetScope program (Net-Base Software GmbH, Freiburg, Germany).

#### 4.5.6. Confocal Laser Scanning Microscopy

Confocal laser scanning microscopy (LSM 510, Zeiss, Göttingen, Germany) was performed to reveal co-localization of hAPP with QC and pE-Abeta. The Cy2-labeled pE-Abeta (green fluorescence) was visualized by excitation at 488 nm and the detection of emission at 510 nm using a low-range band pass (505–530 nm). The Cy3-labeled hAPP (red fluorescence) was visualized using excitation at 543 nm and emission at 570 nm, and the Cy5-labeled QC (blue fluorescence) was detected using excitation at 650 nm and emission at 670 nm. Antibody specificity was confirmed by omitting primary antibodies. Photoshop CS2 (Adobe Systems, Mountain View, CA, USA) was used to process the images obtained by light and confocal laser scanning microscopy with minimal alterations to brightness, sharpness, color saturation, and contrast.

## Figures and Tables

**Table 1:** Spatial expression of hAPP and endogenous QC and deposition of pE-Abeta in Tg2576 brain.

Brain Region	hAPP	Endogenous QC	pE-Abeta
anterodorsal thalamic nucleus	4–5	0	0
Edinger-Westphal nucleus	5	5	0
locus coeruleus	3	5	0
hippocampus			
granule cells	0	0	0
interneurons	4	3	3
pyramidal cells	1	1	1
piriform cortex	4–5	3	5
amygdala	5	2	3
lateral hypothalamus	2	4–5	0
perifornical nucleus	4	0	0

## Abbreviations

AD	Alzheimer’s disease
AP	alkaline phosphatase
APP	amyloid precursor protein
BSA	bovine serum albumin
DAB	3,3′-Diaminobenzidine
ITC	isothermal titration calorimetry
NOS	nitric oxide synthase
TBS	tris-buffered saline
pE	pyroglutamate
QC	glutaminyl cyclase
